# CAMKK2-AMPK axis endows dietary calcium and phosphorus levels with regulatory effects on lipid metabolism in weaned piglets

**DOI:** 10.1186/s40104-024-01061-0

**Published:** 2024-08-05

**Authors:** Zhenyan Miao, Yanjie Sun, Zhangjian Feng, Qiwen Wu, Xuefen Yang, Li Wang, Zongyong Jiang, Ying Li, Hongbo Yi

**Affiliations:** 1 State Key Laboratory of Swine and Poultry Breeding Industry, Key Laboratory of Animal Nutrition and Feed Science in South China, Ministry of Agriculture and Rural Affairs, Maoming Branch, Guangdong Laboratory for Lingnan Modern Agriculture, Guangdong Provincial Key Laboratory of Animal Breeding and Nutrition, Institute of Animal Science, Guangdong Academy of Agricultural Sciences, Guangzhou, 510642 China; 2 College of Veterinary Medicine, South China Agricultural University, Guangzhou, 510642 China

**Keywords:** AMPK, CAMKK2, Dietary calcium and phosphorus, Intestine-liver axis, Lipid metabolism

## Abstract

**Background:**

In the realm of swine production, optimizing body composition and reducing excessive fat accumulation is critical for enhancing both economic efficiency and meat quality. Despite the acknowledged impact of dietary calcium (Ca) and phosphorus (P) on lipid metabolism, the precise mechanisms behind their synergistic effects on fat metabolism remain elusive.

**Results:**

Research observations have shown a decreasing trend in the percentage of crude fat in carcasses with increased calcium and phosphorus content in feed. Concurrently, serum glucose concentrations significantly decreased, though differences in other lipid metabolism-related indicators were not significant across groups. Under conditions of low calcium and phosphorus, there is a significant suppression in the expression of FABPs, CD36 and PPARγ in the jejunum and ileum, leading to inhibited intestinal lipid absorption. Concurrently, this results in a marked increase in lipid accumulation in the liver. Conversely, higher levels of dietary calcium and phosphorus promoted intestinal lipid absorption and reduced liver lipid accumulation, with these changes being facilitated through the activation of the CAMKK2/AMPK signaling pathway by high-calcium-phosphorus diets. Additionally, the levels of calcium and phosphorus in the diet significantly altered the composition of liver lipids and the gut microbiota, increasing α-diversity and affecting the abundance of specific bacterial families related to lipid metabolism.

**Conclusion:**

The evidence we provide indicates that the levels of calcium and phosphorus in the diet alter body fat content and lipid metabolism by modulating the response of the gut-liver axis to lipids. These effects are closely associated with the activation of the CAMKK2/AMPK signaling pathway.

**Supplementary Information:**

The online version contains supplementary material available at 10.1186/s40104-024-01061-0.

## Introduction

Calcium and phosphorus, fundamental to the mineral nutrient spectrum, play an indispensable role in bone development and act as critical secondary messengers in cellular signaling. The significance of these minerals transcends the mere provision of skeletal integrity, extending to a broad spectrum of physiological processes, including muscle contraction, the facilitation of neurotransmitter dissemination, hormone secretion, and the regulation of body weight equilibrium. These roles underscore the indispensable contribution of calcium and phosphorus to both developmental and homeostatic mechanisms within the biological system [1, 2]. In the field of lipid metabolism, calcium and phosphorus are essential for the maintenance of a healthy state. Calcium augments energy utilization and weight regulation through the facilitation of lipolysis, amplification of insulin sensitivity, meticulous regulation of fatty acid oxidation, and nuanced modulation of cholesterol levels. On the other hand, phosphorus occupies a central position in energy metabolism. The role of phosphorus in ATP production is well-established, significantly impacting lipid synthesis and catabolism, lipoprotein metabolism, and, through the regulation of hormones like insulin, subsequently influencing the overarching lipid equilibrium. Calcium supplementation has been shown to mitigate organ fat accumulation induced by a high-fat diet and to enhance intramuscular fat storage in livestock, indicating its potential in improving meat quality and animal health [3]. Conversely, whilst phosphorus supplementation was able to reduce overall lipid content, it also reduced intramuscular fat content, demonstrating its subtle effects on lipid distribution within muscle tissue [1, 4]. Previous studies have focused on the dual regulation of calcium and phosphorus on muscle performance, leaving a gap in understanding the effects of dietary calcium and phosphorus changes on overall lipid metabolism and the mechanisms involved. This knowledge gap underscores the need for a more comprehensive exploration of how these minerals interact in complex networks of lipid metabolism that may provide new nutritional strategies for animal health and production performance.

In the animal body, lipids play a multifaceted and critical role, not only as a major energy reservoir, but also influencing livestock production and meat quality. However, excessive accumulation of fat not only reduces lean meat percentage and meat quality, but also affects feed conversion efficiency and may even lead to serious health problems such as fatty liver [5]. Therefore, understanding and controlling fat accumulation and its metabolic processes are important for improving meat quality, enhancing animal health, and improving feed efficiency. As a central regulator of intracellular energy metabolism, 5′-adenosine monophosphate (AMP)-activated protein kinase (AMPK) is essential for maintaining the stable availability of glucose, glycogen and fatty acids. In addition, AMPK plays a key role in signaling pathways that sense intracellular lysosomal and nuclear DNA damage [6]. Studies have shown that AMPK has an important role in the regulation of lipid and glucose metabolism in the heart, hypothalamus, adipose tissue, muscle, and liver. Calcium has a marked inhibitory effect on endogenous lipid production in the liver through activation of the AMPK pathway, but the deeper molecular mechanisms have not been elucidated [7]. As an upstream signal of AMPK, calcium/calmodulin-dependent kinase kinase 2 (CAMKK2) can enhance its regulatory effect on lipid metabolism by activating AMPK, and conversely AMPK activation can influence Ca^2+^ signaling and modulate CAMKK2 activity. The function of CAMKK2, especially in regulating lipid metabolism has been demonstrated in several studies [8–10]. Both genetic deletion and pharmacological inhibition of CAMKK2 significantly reduces de novo fatty acid synthesis and may bring about amelioration of high-fat diet-induced fatty liver, reduced insulin sensitivity, and prostate cancer cell proliferation [11, 12].

Although calcium and phosphorus, as key mineral nutrients, play important roles in maintaining the body’s lipid metabolism homeostasis, the understanding of how different dietary calcium and phosphorus intakes specifically affect lipid metabolism and their molecular mechanisms is incomplete. In view of this, the aim of this study was to investigate the effects of different calcium and phosphorus levels (normal, low and high) on lipid metabolism and the mechanisms behind them using a weaned piglet model. In particular, this study focused on the interaction between intestine and liver during lipid absorption and processing, revealing how increased calcium and phosphorus levels ameliorate disordered lipid metabolism by activating the AMPK/CAMKK2 pathway in the intestine-liver axis. Further, this study also examined the correlation between gut microbiota and liver lipid composition, exploring how the intestine-liver axis serves as a key mechanism by which dietary calcium and phosphorus regulate lipid metabolism. This comprehensive study not only deepens our understanding of the role of calcium and phosphorus in the regulation of lipid metabolism, but also provides new insights into how to optimize lipid metabolism by modulating calcium and phosphorus intake in the diet.

## Materials and methods

### Ethics statement

All animal experimental procedures were approved by the Laboratory Animal Welfare Ethics Committee of the Institute of Animal Science, Guangdong Provincial Academy of Agricultural Sciences, in accordance with the current animal protection law (Ethical approval code: GDIAS20221103).

### Animals and experimental protocol

Seventy-two weaned piglets (Duroc × Landrace × Large White), with an initial body weight of 7.23 ± 0.92 kg and aged 25 d, were randomly assigned to 3 treatment groups. Each treatment included 6 replicate pens, with each pen housing 4 piglets (2 castrated males and 2 females). No significant differences in body weight were observed between pens (*P* > 0.05). The experiment was divided into two phases: the 7–11 kg phase and the 12–25 kg phase. The study demonstrated that weaned piglets exhibited higher average daily gain and feed conversion ratio when the dietary ratio of standardized total tract digestible (STTD) calcium to STTD phosphorus was 1.2 [13]. Therefore, the NRC (2012) [14] recommended STTD P levels (0.34% to 0.42%) were used as the control group, with the difference in the recommended STTD P levels between the two phases serving as the concentration gradient. Accordingly, this study adjusted the dietary calcium and phosphorus levels based on the NRC (2012) [14] nutritional guidelines to maintain a constant STTD Ca to STTD P ratio of 1.2. The STTD Ca:STTD P ratios for the 3 groups were 0.504%:0.42% (CON), 0.216%:0.18% (LCAP), and 0.696%:0.58% (HCAP). The specific dietary composition and nutritional levels are detailed in Table 1. Additionally, to eliminate the influence of phytase on the experiment, no phytase was added to the diets. The feed was prepared as pelleted feed, with pelleting temperatures ranging from 80 to 83 °C and a die aperture diameter of 3.5 mm. For the first phase, 200 kg of feed was produced per batch, while 135 kg was produced per batch for the second phase. Animals were housed in individual pens of equal size. The trial period lasted for 42 d, during which the piglets had ad libitum access to feed and were provided with ample clean water. All pigs were vaccinated according to standard immunization protocols.

**Table 1:** Compositions and nutrient levels of experimental diets (air-dried basis), %

Items	7 – 11 kg phase			12 – 25 kg phase		
	CON	LCAP	HCAP	CON	LCAP	HCAP
Corn	29.51	29.51	29.51	52.82	52.82	52.82
Puffed corn	25.00	25.00	25.00	10.00	10.00	10.00
Soybean meal	16.97	16.97	16.97	22.64	22.64	22.64
Low protein whey powder	10.00	10.00	10.00	3.00	3.00	3.00
Whey protein concentrate^a^	10.00	10.00	10.00	3.00	3.00	3.00
Soybean oil	2.00	2.00	2.00	2.00	2.00	2.00
50% Choline chloride	0.20	0.20	0.20	0.20	0.20	0.2
Sodium chloride	0.40	0.40	0.40	0.40	0.40	0.40
L-Lysine	0.37	0.37	0.37	0.46	0.46	0.46
DL-Methionine	0.14	0.14	0.14	0.14	0.14	0.14
L-Threonine	0.07	0.07	0.07	0.13	0.13	0.13
L-Valine	-	-	-	0.04	0.04	0.04
Titanium dioxide	0.40	0.40	0.40	0.40	0.40	0.40
Zinc oxide	0.20	0.20	0.20	-	-	-
Mineral premix^b^	1.00	1.00	1.00	1.00	1.00	1.00
Limestone	1.02	0.42	1.41	1.05	0.47	1.43
Calcium dihydrogen phosphate	1.12	0.10	1.80	1.24	0.2	1.94
Sodium dihydrogen phosphate	0.25	0.05	0.37	0.28	0.11	0.38
Sodium bicarbonate	0.23	0.36	0.16	0.08	0.19	0.02
Silicon dioxide	1.12	2.81	0.00	1.12	2.80	0.00
Total	100.00	100.00	100.00	100.00	100.00	100.00
Nutrient levels^c^						
NE, kcal/kg	2,553	2,553	2,553	2,500	2,500	2,500
CP	21.06	20.90	21.05	19.01	19.02	19.01
SID Lys	1.48	1.48	1.48	1.27	1.27	1.27
SID Thr	0.87	0.87	0.87	0.75	0.75	0.75
SID Met	0.47	0.47	0.48	0.41	0.41	0.41
SID Tyr	0.28	0.28	0.28	0.21	0.21	0.21
SID Val	0.93	0.92	0.93	0.93	0.93	0.93
Ca	0.626	0.33	0.882	0.638	0.41	0.894
Total P	0.594	0.291	0.762	0.594	0.33	0.732
STTD Ca	0.504	0.216	0.696	0.504	0.216	0.696
STTD P	0.42	0.18	0.58	0.42	0.18	0.58

^a^Concentrated whey protein powder is an uncommon raw material, containing 0.25% Ca, 0.26% P, 2,797 kcal/kg of net energy, 76.32% crude protein, 1.33% crude fiber, and 0.2% crude fat. Its STTD of Ca is 88.00%, STTD of P is 92.00%, STTD Ca is 0.30%, STTD P is 0.45%

^b^The premix provides a full diet per kilogram: vitamin A, 9,920 IU; vitamin D_3_, 2,240 IU; vitamin E, 24 mg; vitamin K_3_, 4 mg; vitamin B_1_, 2.4 mg; vitamin B_2_, 8 mg; vitamin B_6_, 6.4 mg; vitamin B_12_, 32 mg; niacin, 32 mg; pantothenic acid, 12 mg; folic acid, 0.8 mg; biotin, 64 mg; iron, 90 mg; copper 12 mg; manganese, 52.5 mg; iodine, 0.525 mg; selenium, 0.36 mg; zinc, 60 mg

^c^Crude protein, calcium, and total phosphorus were analyzed values, the rest were calculated values

### Sample collection

After a 12-h fast starting at 20:00 on d 42 of the trial, each piglet was weighed individually at 8:00 on d 43. From each group, 3 female piglets and 3 castrated male piglets closest to the group’s average body weight were selected, totaling 6 piglets per group and 18 piglets in all. Prior to slaughter, 10 mL of blood was collected from the anterior vena cava of each of the 18 selected piglets into anticoagulant tubes, and an additional 10 mL was collected into non-anticoagulant tubes and allowed to stand for 1 h. The samples were then centrifuged at 3,500 r/min and 4 °C for 12 min to separate serum and plasma. These were aliquoted into sterile 1.5 mL EP tubes, with each tube containing 500 μL, and stored at −20 °C for subsequent biochemical analysis of serum indicators. The piglets were anesthetized using electrical stunning after blood collection, followed by euthanasia. Upon opening the abdominal cavity, 1 cm ring-shaped sections from the mid-jejunum and distal ileum, as well as 3 cm^3^ of liver tissue, were collected. These samples were gently rinsed with phosphate-buffered saline (PBS) using a syringe, then quickly frozen in liquid nitrogen and stored at −80 °C for subsequent research. Additionally, chyme from the colon was collected, rapidly frozen in liquid nitrogen, and stored at −80 °C for further experiments. Portions of the liver and intestinal tissues were fixed in 4% paraformaldehyde for subsequent histological examination.

### Carcass chemical analysis

In summary, on d 43 of the experiment, after collecting the required samples from each slaughtered piglet, all remaining blood, intestinal and liver tissues, and other abdominal organs, along with the entire body, were processed into a bone and meat mixture using grinders of various sizes. The chopped samples were thoroughly homogenized in a blender. Wet samples were taken using the quartering method, spread evenly on petri dishes, dried in an oven at 65 °C for 72 h, vacuum-packed, and stored at −20 °C until further analysis. The total fat content of carcass was determined by Soxhlet extraction method. Briefly, three replicates of each sample were taken and about 30 g of carcass samples were ground into minced meat, dried in a vacuum freeze dryer and then ground into powder. 1 g of dried meat sample (accurate to 0.0001 g) was wrapped in filter paper into a cylindrical filter cup and extracted with n-hexane at 140 °C for 50 min in a Soxhlet extraction unit (SE-A6, ALVA, Jinan, China). After air-drying for 10 min and baking at 102 °C for 30 min, the extracted oil-containing aluminium cups were accurately weighed (to the nearest 0.0001 g) when the aluminium cups were cooled to room temperature. Total carcass fat (%) = (weight of oil-containing aluminium cups after extraction − weight of empty aluminium cups)/weight of air-dried meat samples × 100%.

### Biochemical analysis

Serum total cholesterol (TC), triglyceride (TG), high-density lipoprotein cholesterol (HDL-C), low-density lipoprotein cholesterol (LDL-C), and glucose (GLU) concentrations were determined using an automated biochemical analyser (iMagic-M7, ICUBIO, Shenzhen, China). Intestinal wall samples, liver samples and carcass samples were homogenised in saline solution (1:9) and sediment was removed by centrifugation (3,000 r/min, 10 min) to obtain 10% tissue homogenate. The TC and TG levels in the intestines, liver and carcass were all measured using colorimetric assay kits (Nanjing Jiancheng Bioengineering Institute, Nanjing, China) according to the manufacturer’s instructions.

### Histological analysis

The liver tissues of piglets fixed in 4% paraformaldehyde were frozen and embedded. The cryosections were cut into 10 μm thickness and stained with Oil red O (G1015, Servicebio, Wuhan, China). The sections were washed with 85% propylene glycol and then with distilled water before being stained with hematoxylin. The staining process was repeated after each wash. The presence of lipid droplets was indicated by a red stain. Sections were examined under a microscope (B80i, Nikon, Tokyo, Japan), photographed and recorded using Image-Pro Plus 6.0 software for subsequent comparison.

### Real-time quantitative PCR analysis

Total RNA from intestine and liver tissues was extracted with TRIzol reagent (Takara Bio, Shiga, Japan). 1 μg of RNA was then reverse transcribed to cDNA using the PrimeScript RT kit with cDNA Eraser (Takara Bio) according to the kit instructions. Quantitative PCR (qPCR) was conducted on a CFX Connect Detection System (Bio-Rad, Hercules, CA, USA) using a SYBR Green mixing kit (Takara Bio, Shiga, Japan) in a reaction volume of 20 μL. The 20 μL of reaction system showed as below, SYBR Premix Ex Taq (10 μL), upstream primer (0.4 μL), upstream primer (0.4 μL), downstream primer (0.4 μL), ROX II dye (0.4 μL), cDNA samples (2 μL), and ultrapure water (6.8 μL).

The real-time quantitative PCR reaction conditions were: pre-denaturation at 95 °C for 5 min, denaturation at 95 °C for 5 s, annealing at 60 °C for 34 s, extension at 95 °C for 15 s, and the number of cycles of amplification was 40. The results were calculated by the 2^−ΔΔCt^ method, and all the primer sequences required for qPCR in this study were shown in Table S1.

### Western blot analysis

The samples were separated by SDS-PAGE and the protein was transferred to PVDF membrane (MilliporeSigma, Burlington, MA, USA). The following antibodies were used in western blotting, such as β-actin (Affinity, T0022, 1:3,000), PPARγ (Abcam, 209350, 1:1,000), SIRT1 (Cell Signaling Technology, 9475S, 1:1,000), AMPKα (Cell Signaling Technology, #5831, 1:1,000), phospho-AMPKα (Thr172; Cell Signaling Technology, #2535, 1:1,000), CAMKK2 (Proteintech, 111549-1-AP, 1:1,000), phospho-CAMKK2 (Ser511) (Cell Signaling Technology, #12818, 1:1,000), DGAT1 (Abcam, ab54037, 1:1,000), ACC1 (Cell Signaling Technology, #4190, 1:1,000), SREPB1C (Abcam, ab28481, 1:1,000), and FASN (Cell Signaling Technology, 3180S, 1:1,000).

### Immunofluorescence staining

Fixed intestinal tissues were embedded in paraffin and cut into 4 μm sections, then dewaxed, rehydrated, and treated in microwave oven with EDTA-containing antigen retrieval buffer (pH 8.0, Beyotime Biotechnology Co., Ltd., Shanghai, China). Afterwards, sections were blocked with 5% fetal bovine serum (Bioss, Beijing, China) for 1 h at room temperature and then incubated overnight (1:500 dilution) at 4 °C with rabbit anti-AMPKα, CD36, FABP4 (Service Bio, Wuhan, China). After rinsing with PBS, they were incubated with Alexa Fluor 555 (BBI, Shanghai, China)-conjugated goat anti-rabbit secondary antibody for 30 min at room temperature in the dark. In addition, images were obtained under a microscope with magnification of 200 (Nikon, Tokyo, Japan) and positive results were quantified using ImageJ 1.54 software.

### Non-targeted lipidomics of liver

The total lipids were extracted from the livers of three groups of piglets. After thawing, liver tissue (20 mg) was homogenized in 1 mL mixture (methanol, MTBE, and internal standard mixture). After that, ultrasound was performed at 4 °C for 20 min and then left at room temperature for 30 min. The solution was centrifuged at 10 °C at 14,000 × *g* for 15 min to obtain an upper organic solvent layer and dried under nitrogen. Samples were analyzed using a high-performance liquid chromatography system (Nexera UHPLC LC-30A, Shimadzu, Kyoto, Japan) and LC separation was performed on a column (Acquity Premiercsh C18, 1.7 μm × 2.1 mm × 100 mm, Waters, Milford, USA). The lipid extract was redissolved in 200 mL of 90% isopropanol/acetonitrile, centrifuged at 14,000 × *g* for 15 min, and finally injected with 3 mL of the sample. Solvent A was acetonitrile–water (6:4, v/v) containing 0.1% formic acid and 0.1 mmol/L ammonium formate, and solvent B was acetonitrile-isopropanol (1:9, v/v) containing 0.1% formic acid and 0.1 mmol/L ammonium formate. The initial flow rate was 300 μL/min with 40% solvent B. Hold for 3.5 min, then linearly increase to 75% of solvent B within 9.5 min, then linearly increase to 99% of solvent B within 6 min, then equilibrate in 40% of solvent B for 5 min. The mass spectra were obtained by Q-exactive Plus in both positive and negative modes. All measured ESI parameters were optimized and preset as follows: source temperature, 300 °C; capillary temperature, 350 °C, ion spray voltage set to 3,000 V, and S-Lens RF level set to 50%, respectively, the scanning range of the instrument is set to *m/z* 200–1,800. Lipid search was used for peak recognition, peak extraction, and lipid identification (secondary identification). Fatty acid composition expressed as a percentage of total fatty acids.

### 16S rRNA sequencing and processing

Total genomic DNA of the gut microbiota was extracted from samples of colon contents using a DNA isolation kit (Omega Bio-Tek, Norcross, GA, USA). DNA concentration was determined using a Nanodrop instrument (Thermo Fisher Scientific, Waltham, MA, USA). DNA integrity was assessed using 2% agar gel electrophoresis. Universal forward primer (5'-CCTAYGGGGRBGCASCAG-3') and reverse primer (5'-GGACTACNNGGGGTATCTAAT-3') were used to amplify the V3-V4 region of the bacterial 16S rRNA gene. Sequencing was performed on the Illumina MiSeq/NovaSeq platform. The DADA2 module in QIIME2 software (Version QIIME2-202202) was used for noise reduction. Microbial composition diversity was analysed using QIIME and R software. PICRUSt2 software was used for KEGG pathway analysis.

### Short-chain fatty acid analysis

Short-chain fatty acids in colon contents were determined using gas chromatography. To prepare the sample, 50 mg of colon contents were mixed with 250 µL of ultrapure water for 5 min. The suspension was then centrifuged at 5,000 r/min for 30 min. Next, 1 mL of supernatant was transferred to a 2-mL PE tube and mixed with 200 μL of 42 mmol/L crotonic acid and 200 μL of 10% metaphosphoric acid solution. The PE tubes were refrigerated overnight at 4 °C and then centrifuged at 10,000 r/min for 10 min at 4 °C. The resulting supernatant was mixed with an equal amount of ether and extracted for 5 min. The ether layer was aspirated using a disposable syringe, filtered through a 0.22-μm organic membrane, and then injected into a brown vial for injection. Short-chain fatty acids (acetic, propionic, butyric, valeric, isobutyric and isovaleric acids, with crotonic acid as an internal standard) were measured using a gas chromatograph and a mass spectrometry detector (7890A and 5975C Inert XL EI/CI Mass Detectors, Agilent Technologies, Santa Clara, CA, USA). Detection of short-chain fatty acids was performed according to a previous GC procedure [15].

### Data analysis

The results were statistically analyzed using SPSS 21.0 statistical analysis software, and the data were expressed as mean ± standard deviation. Statistical treatment was performed using one-way analysis of variance (ANOVA) followed by LSD post-test. *P* < 0.05 was considered statistically significant.

## Results

### Effect of calcium and phosphorus content on lipid homeostasis

**Fig. 1:**
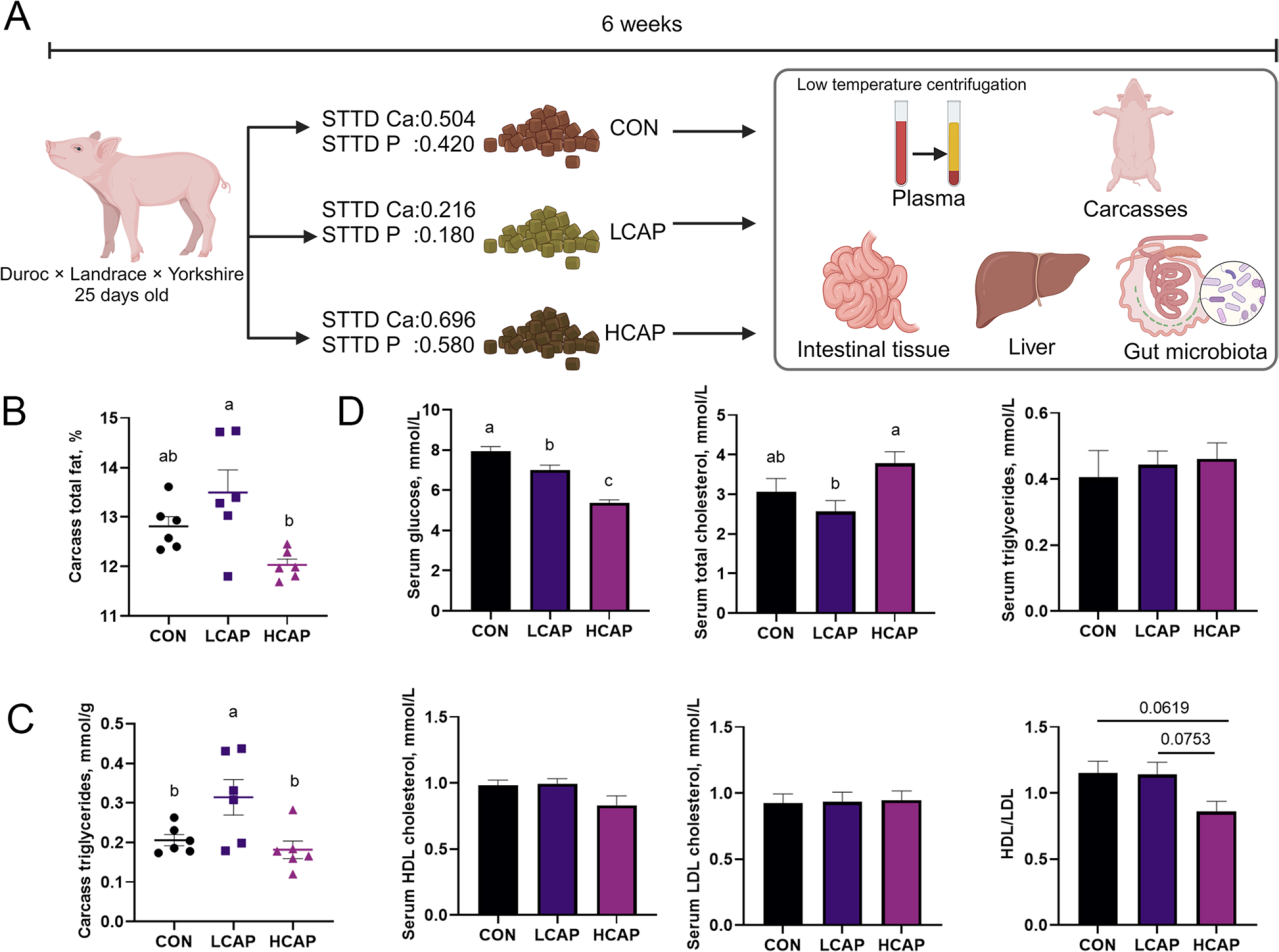
Dietary calcium and phosphorus levels affect carcass and blood lipids. **A** Experimental procedure. Created with BioRender.com. **B** Percentage of total fat in carcass. **C **Total triglyceride content in carcass. **D** Serum glucose, total triglyceride, total cholesterol, HDL-C, and LDL-C levels, and the ratio of HDL-C to LDL-C. Data are means and standard errors for 6 pigs per treatment. Different letters indicate significant differences (*P* < 0.05). CON, control group; LCAP, low-calcium-phosphorus group; HCAP, high-calcium-phosphorus group

In a six-week dietary intervention study (Fig. 1A), we systematically evaluated the effects of dietary calcium and phosphorus on lipid homeostasis in piglets. We first measured the percentage of carcass crude fat and total triglyceride levels to assess the direct impact of dietary calcium and phosphorus on lipid accumulation. The results showed that, compared to the LCAP group, the HCAP group had significantly lower percentages of carcass crude fat (Fig. 1B) and total carcass triglycerides (Fig. 1C) (*P* < 0.05). Further analysis of piglet serum biochemical parameters (Fig. 1D) revealed that piglets fed the high calcium and phosphorus diet had significantly lower serum glucose concentrations than the LCAP group (*P* < 0.05), while their serum total cholesterol concentrations were significantly higher. Other lipid metabolism-related indicators showed no significant differences among the 3 groups.

### Dietary calcium and phosphorus content regulates intestinal lipid absorption and deposition

**Fig. 2:**
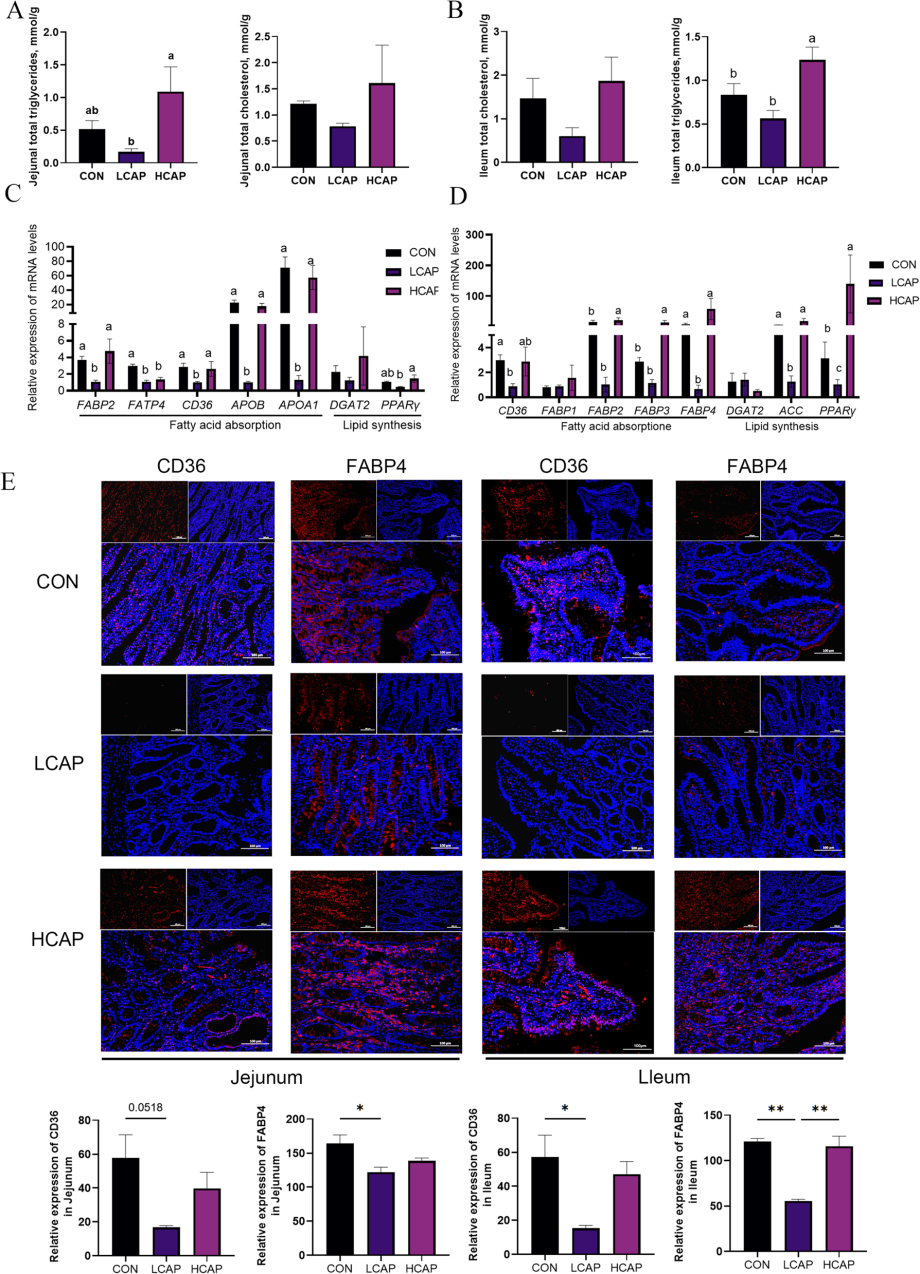
Effect of dietary calcium and phosphorus levels on intestinal lipid absorption. **A** and **B** Total triglyceride and total cholesterol levels in the jejunum ileum. **C** and **D** Lipid transport (*CD36*, *FABP1*, *FABP2*, *FABP3*, *FABP4*, *APOA1*, *APOB*) and lipid synthesis (*DGAT2*, *ACC*, *PPARγ*) gene expression abundance in the jejunum and ileum. Data are means and standard errors for 6 pigs per treatment. Different letters indicate significant differences (*P* < 0.05). CON, control group; LCAP, low calcium and phosphorus group; HCAP, high calcium and phosphorus group; CD36, cluster of differentiation 36; FABP1, fatty acid binding protein 1; FABP2, fatty acid binding protein 2; FABP3, fatty acid binding protein 3; FABP4, fatty acid binding protein 4; APOA1, apolipoprotein A1; APOB, apolipoprotein B; DGAT2, diacylglycerol O-acyltransferase 2; ACC, acetyl CoA carboxylase; PPARγ, peroxisome proliferator-activated receptor gamma

By measuring the lipid content of the jejunum and ileum (Fig. 2A and B), we found that the levels of triglyceride and cholesterol within the intestinal tissues were proportional to the Ca and P content in the feed, particularly in the HCAP group, the levels of triglyceride in jejunum and ileum were significantly increased (*P* < 0.05). Further exploring the regulation of intestinal lipid metabolic pathways, we analyzed the expression of key lipid metabolism genes. The results showed that LCAP significantly inhibited the expression of lipid transport and absorption related genes such as *FABP2*, *CD36*, *APOB*, *APOA1*, as well as *FABP3* and *FABP4* in jejunum and ileum (Fig. 2C and D; *P* < 0.05). Notably, both high and low calcium-phosphorus diets appeared to suppress *FATP4* expression in the jejunum. Meanwhile, low calcium-phosphorus levels also significantly suppressed the expression of fat synthesis-related genes, such as *PPARγ* and *ACC* (*P* < 0.05), whereas *DGAT2* expression tended to decrease but did not reach a significant level. By immunofluorescence technique, we further validated the expression profiles of CD36 and FABP4 proteins in jejunum and ileum (Fig. 2E), consistent with the results of gene expression analysis. These results together revealed that dietary calcium and phosphorus content had significant effects on intestinal lipid absorption and metabolism.

### Dietary calcium and phosphorus levels regulate intestinal lipid absorption through AMPK/CAMKK2 pathway

**Fig. 3:**
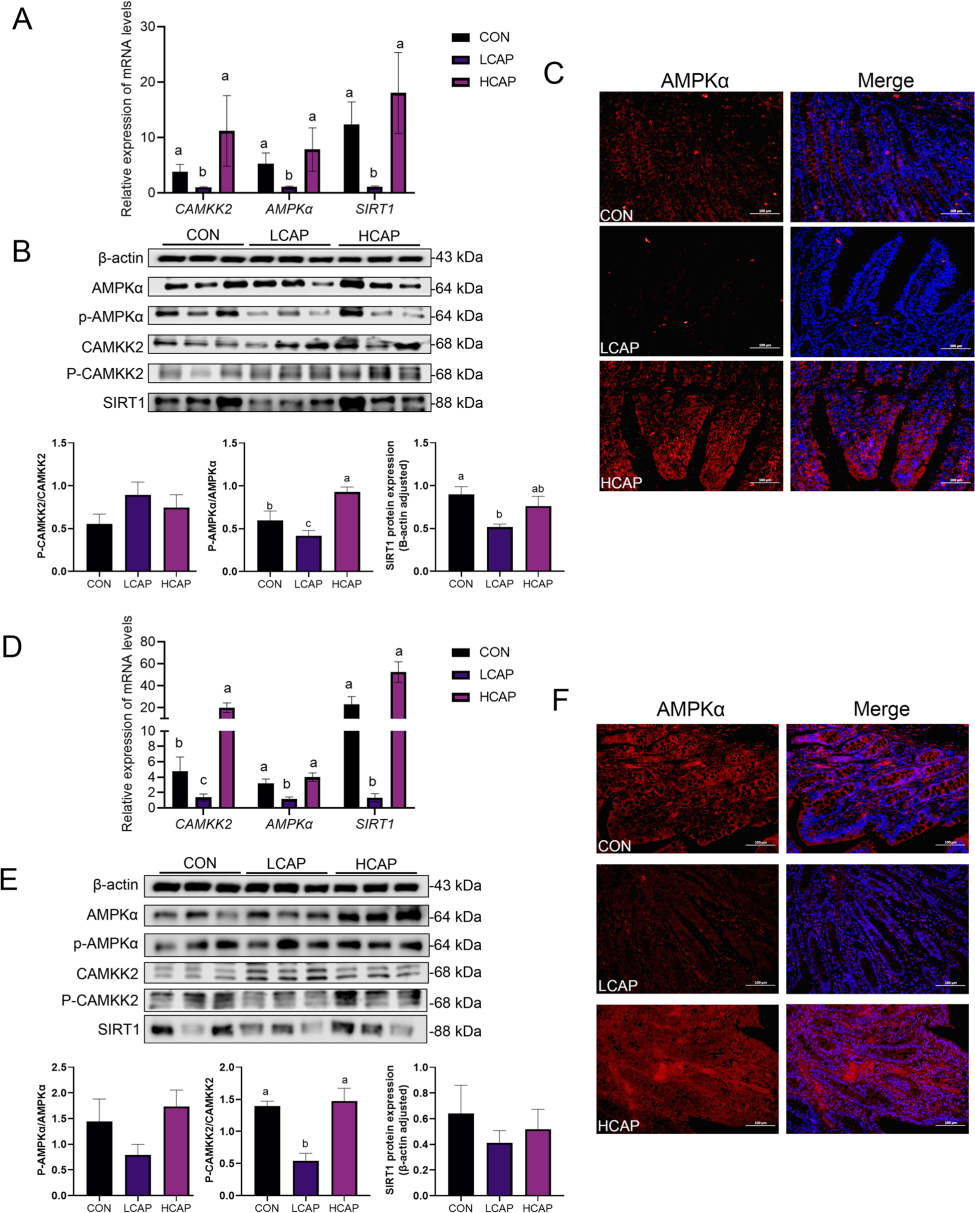
Dietary calcium and phosphorus levels regulate intestinal lipid metabolism through the CAMKK2/AMPK pathway. **A** Expression abundance of *AMPKα* and its upstream and downstream genes in the jejunum. **B** Quantification of AMPKα signaling-associated protein bands and protein expression abundance in the jejunum and monitoring with β-actin. **C** Immunofluorescence validation of jejunal AMPKα protein. **D** Expression abundance of *AMPKα* and its upstream and downstream genes in the ileum. **E** Quantification of AMPKα signaling-associated protein bands and protein expression abundance in ileum and monitoring with β-actin. **F** Immunofluorescence validation of ileal AMPKα protein. Data are means and standard errors for 6 pigs per treatment. Different letters indicate significant differences (*P* < 0.05). CON, control group; LCAP, low calcium-phosphorus group; HCAP, high calcium-phosphorus group; AMPKα, AMP-activated protein kinase alpha; CAMKK2, calcium/calmodulin-dependent protein kinase kinase 2; SIRT1, sirtuin 1

In order to study the mechanism of calcium and phosphorus regulating lipid absorption, several key parameters of AMPK/CAMKK2 pathway were measured. The expression of *AMPKα* and its upstream and downstream regulatory genes in the jejunum was significantly suppressed in the low-calcium-phosphate diet group (Fig. 3A; *P* < 0.05). The western blot analysis indicated that the low-calcium-phosphorus diet significantly suppressed the protein levels of phosphorylated AMPKα (P-AMPKα) and sirtuin 1 (SIRT1) in the jejunum (Fig. 3B; *P* < 0.05). However, there was no significant difference in the level of phosphorylation of CAMKK2 (P-CAMKK2). The results of immunofluorescence were consistent with the immunoblot analysis, further validating this finding. The results of ileal analysis also supported these findings, showing that the expression of *AMPK* and its upstream and downstream genes was significantly suppressed under low calcium-phosphorus conditions (Fig. 3D; *P* < 0.05). The low calcium-phosphorus diet resulted in a significant decrease in P-AMPKα protein levels (Fig. 3E and F; *P* < 0.05), whereas the expression of P-CAMKK2 and SIRT1 proteins showed a decreasing trend but did not reach statistical significance.

### Dietary calcium and phosphorus content regulates liver lipid deposition via the AMPK pathway

**Fig. 4:**
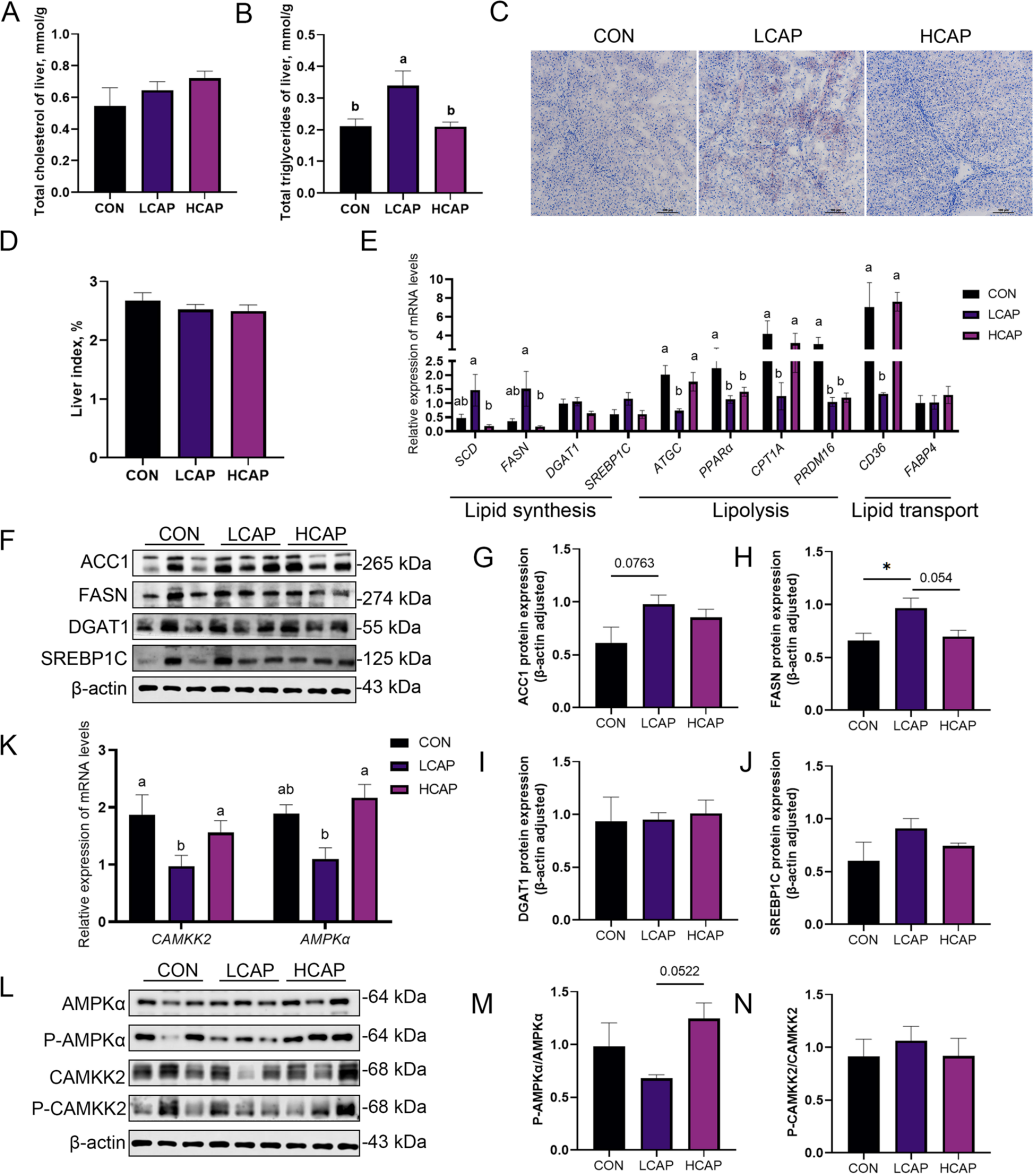
Dietary calcium and phosphorus content regulates liver lipid metabolism through the CAMKK2/AMPK pathway. **A** Liver total cholesterol content. **B** Liver total triglyceride content. **C** Oil red O staining showing liver lipid accumulation. **D** Liver index. **E** Liver lipid synthesis (*SCD*, *FASN*, *DGAT1*, *SREPB1C*), lipid hydrolysis and oxidation (*ATGC*, *PPARα*, *CPT1A*, *PRDM16*), and lipid transport (*CD36*, *FABP4*) gene expression abundance. **F** Liver lipid synthesis-related protein bands. **G**–**J** Protein expression associated with liver lipid synthesis. **K** Liver CAMKK2/AMPK pathway gene expression abundance. **L**–**N** Protein levels of p-AMPK and p-CAMKK2 were examined by protein blot analysis and monitored with AMPK and CAMKK2, respectively. Data are means and standard errors of 6 pigs per treatment; different letters indicate significant differences (*P* < 0.05); CON, control; LCAP, low calcium-phosphorus group; HCAP, high calcium-phosphorus group; SCD, stearoyl-CoA desaturase; FASN, fatty acid synthase; DGAT1, diacylglycerol O-acyltransferase 1; SREBP1C, sterol regulatory element-binding protein 1C; ATGC, Acyl-CoA thioesterase 4; PPARα, peroxisome proliferator-activated receptor alpha; CPT1A, carnitine palmitoyltransferase 1A; PRDM16, PR domain containing 16; CD36, cluster of differentiation 36; FABP4, fatty acid-binding protein 4

We first determined the effect on liver TG deposition and showed that low calcium and phosphorus significantly increased TG accumulation in the liver (Fig. 4A; *P* < 0.05), but had no significant effect on TC levels (Fig. 4B). Oil red O staining further confirmed a significant increase in fat deposition in the liver of piglets in the LCAP group (Fig. 4C), although liver index did not show a statistical difference (Fig. 4D). To deeply explore the effects of calcium and phosphorus levels on the regulatory mechanisms of liver lipid metabolism, we analyzed the gene expression of key enzymes and transcriptional regulators of lipid metabolism in the liver. Low calcium and phosphorus levels significantly up-regulated the expression of lipid synthesis-related genes *SCD* and *FASN* (Fig. 4E; *P* < 0.05), while *DGAT1* and *SREBP1C* showed no significant changes. Meanwhile, the expression of lipolysis-related genes was inhibited and the expression of lipid transport gene *CD36* was significantly decreased (*P* < 0.05). Immunoblotting results were consistent with gene expression analysis showing that low calcium and phosphorus levels promoted the expression of FASN protein (Fig. 4F and H), whereas ACC1 and SREBP1-C tended to be elevated (Fig. 4G and J). There was no significant change in DGAT1 protein expression (Fig. 4I).

### Dietary calcium and phosphorus content alters liver lipid composition

**Fig. 5:**
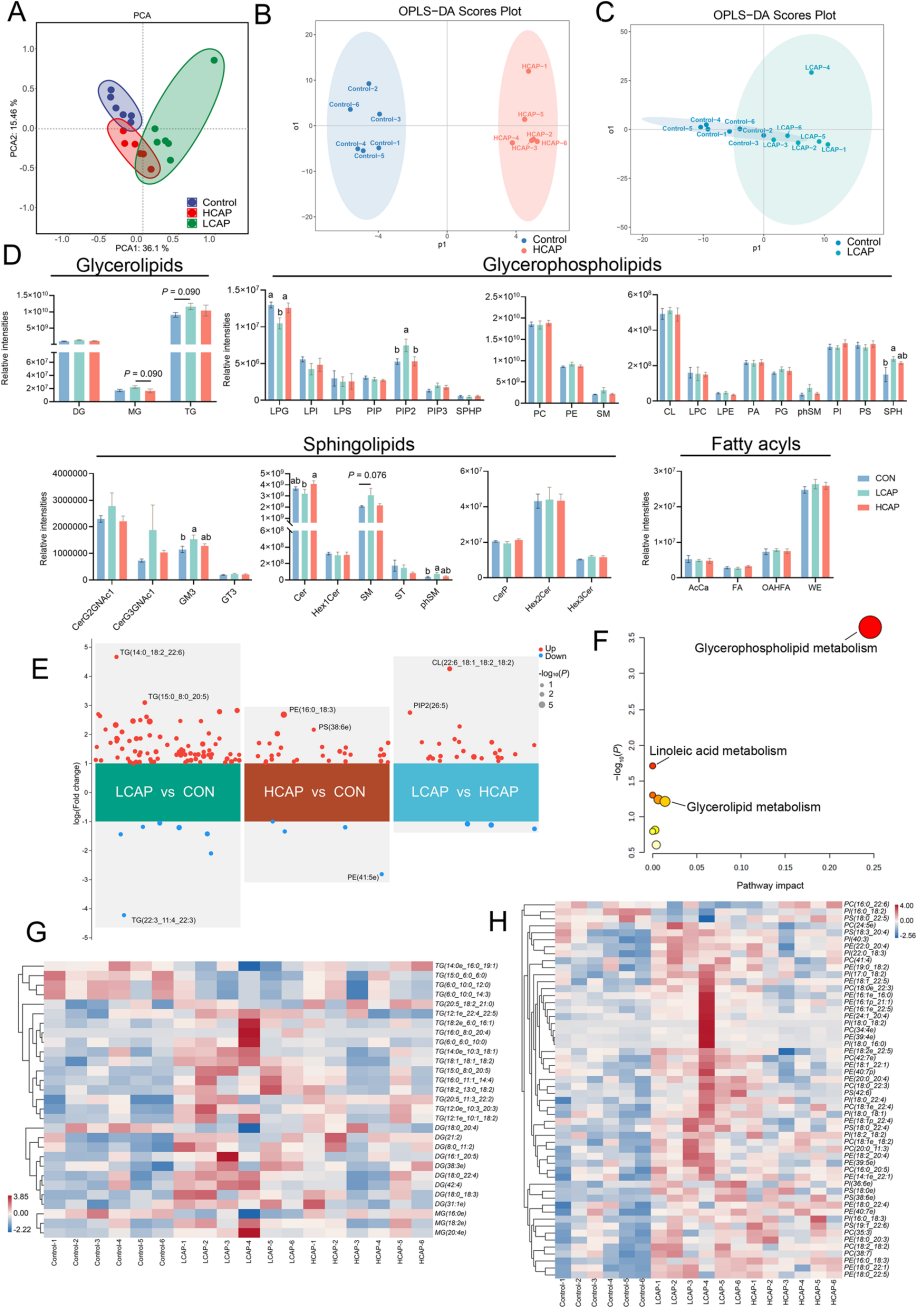
Liver untargeted lipidomic analysis. **A** PCA analysis. (**B** and **C**) OPLS-DA analysis of HCAP vs. CON group and LCAP vs. CON group. **D** Content of glycerolipids, glycerophospholipids, sphingolipids, and fatty acyl groups in CON, LCAP, and HCAP groups; *n* = 6; error lines represent SEM. **E** Volcano plots of two-by-two comparisons between the three groups. **F** Metabolic pathway analysis of differential lipids using MetaboAnalyst. **G** Heat map of relative abundance of glycerol ester differential lipids in the three groups. **H** Heat map of relative abundance of glycerophospholipid differential lipids in the three groups. Data are means and standard errors for 6 pigs per treatment; different letters indicate significant differences (*P* < 0.05)

In this study, we report for the first time the effect of dietary calcium and phosphorus levels on liver lipidomic characteristics. Using UPLC-MS non-targeted lipidomic analysis, we detected more than 45 different lipid classes and 3,429 different lipid molecules in 18 liver samples from three groups (6 samples per group). By principal component analysis (PCA), we observed significant differences in liver lipid metabolites among the three groups (Fig. 5A).

Further orthogonal partial least squares discriminant analysis (OPLS-DA) revealed that the changes of calcium and phosphorus levels had significantly different effects on liver lipid profiles, this is evident from the predicted separation of principal component 1 (Fig. 5B and C). At the lipid class level, different calcium and phosphorus levels in feeds resulted in changes in the content of specific lipid classes (Fig. 5D). We found a trend of increased monoradylglycerols (MG) and TG content in the glyceride category in the LCAP group compared with the CON group (*P* = 0.09). In the glycerophospholipid category, the levels of phosphatidylinositol 4,5-bisphosphate (PIP_2_) and sphingosine (SPH) were significantly increased, while lysophosphatidylglycerol (LPG) was significantly decreased (*P* < 0.05). In the category of nerve sphingolipids, monosyalilated ganglioside M3(GM3) was significantly increased (*P* < 0.05), while the content of SM also tended to increase (*P* = 0.076). We further analyzed differences in lipid metabolites among the three groups using OPLS-DA VIP > 1 and *P*-value < 0.05 as screening criteria for significant differences. The results showed that there were 31 differential lipid metabolites in the LCAP group compared with the control group, 22 differential lipid metabolites in the HCAP group compared with the control group, and 8 differential lipid metabolites in the LCAP group compared with the HCAP Group (Fig. 5E). By analyzing the KEGG pathway for these differential lipid metabolites, we found that calcium and phosphorus levels significantly affected both the glycerophospholipid pathway and the glyceride pathway (Fig. 5F). The analysis of lipids showing significant differences between the glyceride pathway (Fig. 5G) and the glycerophospholipid pathway (Fig. 5H) as shown in the heatmap further confirmed the elevation of the glyceride and glycerophospholipid categories in the LCAP group; This suggests that low levels of calcium and phosphorus disrupt liver lipid metabolism.

### Dietary calcium and phosphorus levels alter gut microbial composition and short-chain fatty acids

**Fig. 6:**
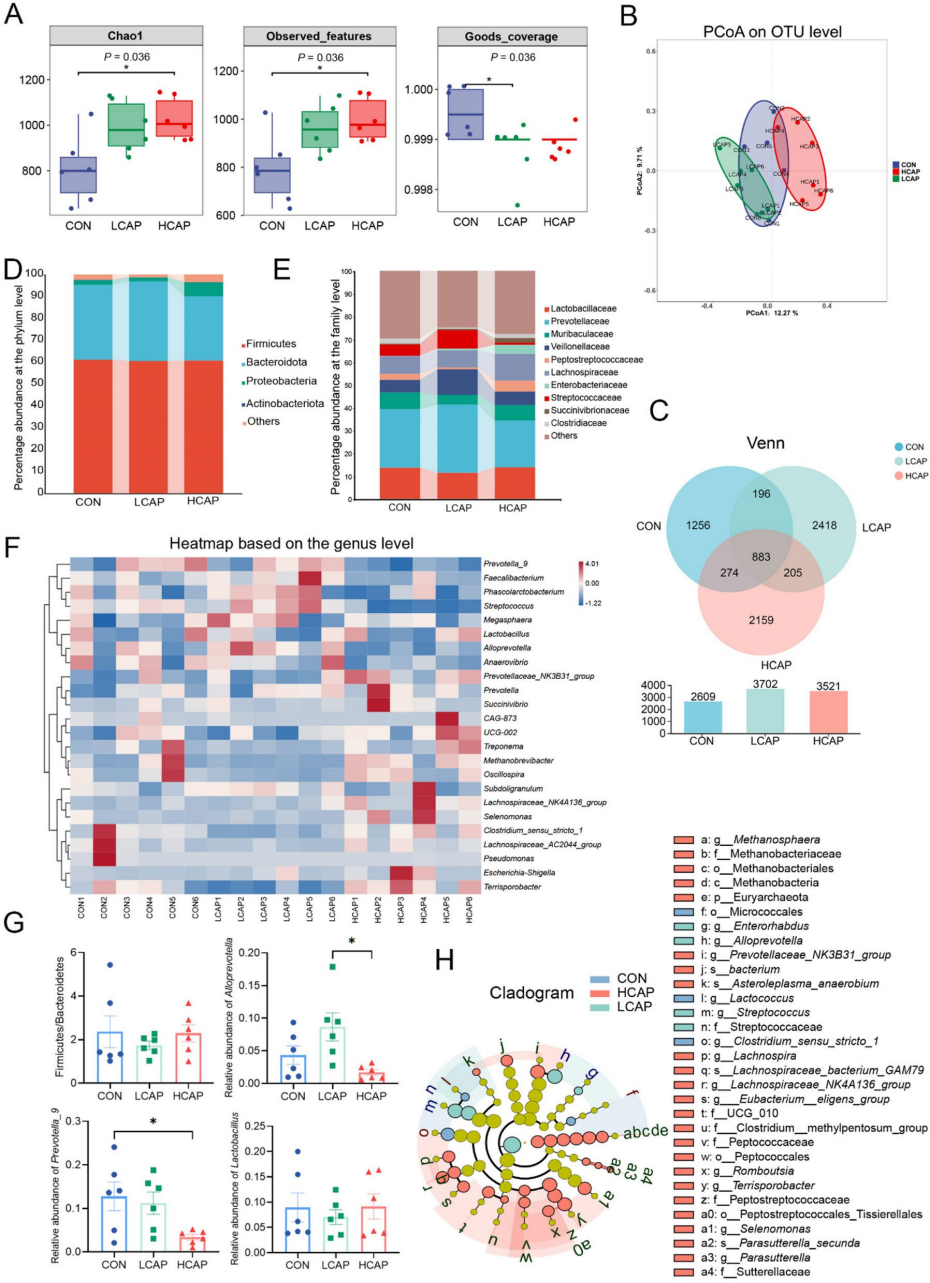
Dietary calcium and phosphorus levels influence the structure of the gut microbiota. **A** α-diversity of the gut flora. **B** β-diversity: principal coordinate analysis (PCoA) based on OTU abundance per weaned piglet. **C** Venn diagram based on OTU levels. **D** and **E** Relative abundance of gut microbiota at the phylum level and family level, respectively. **F** Heat map based on genus level. **G** Changes in flora associated with lipid metabolism: level of Firmicutes/Bacteroidetes, *Alloprevotella* abundance, *Prevotella_9* abundance, *Lactobacillus* abundance. **H** Linear discriminant analysis of effect size (LEfSe) from gate level to genus level (LDA > 3.0). Data are expressed as mean ± SEM and compared with one-way analysis of variance (ANOVA) by Tukey’s multiple comparisons post-test

In order to explore the effects of different dietary calcium and phosphorus levels on the intestinal microbial composition of weaned piglets, we collected colonic content samples from CON, LCAP and HCAP groups, and analyzed them against the bacterial 16S rDNA V3–V4 region using high-throughput sequencing to assess the effects of calcium and phosphorus levels on the structure of intestinal microbial communities. The results of α diversity analysis showed that the CHAO1 index and observed features index in both HCAP and LCAP groups increased, while the goods coverage index decreased, it was suggested that changes in calcium and phosphorus levels in feed might have increased the diversity of gut microbes (Fig. 6A).

**Fig. 7:**
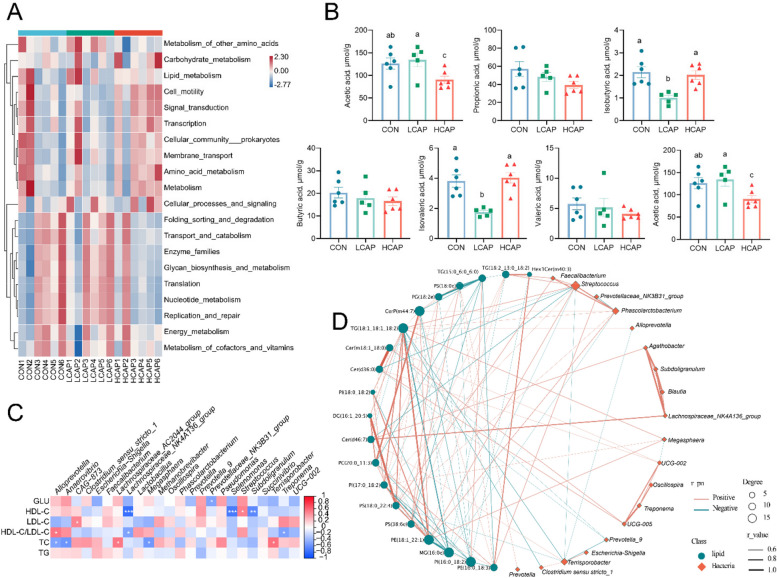
Link between microbiome and lipidomics at different dietary calcium and phosphorus levels. **A** Heat map showing the gut microbiota predicting changes in KEGG pathways at different calcium and phosphorus levels. **B** SCFA (*n* = 6 per group) including acetic acid, propionic acid, isobutyric acid, butyric acid, isovaleric acid pentanoic acid, hexanoic acid, and total short-chain fatty acids were determined in the colonic contents of weaned piglets by gas chromatography; different letters indicate significant differences (*P* < 0.05). **C** Heatmap showing Spearman correlation analysis between gut microbiota and serum lipid metabolism indices. Red represents positive correlations and blue represents negative correlations. Significant correlations are marked with * (*P* < 0.05), ** (*P* < 0.01) and *** (*P* < 0.001). **D** Relationship between discriminatory colonic contents microbial OTUs and significantly different lipid molecules at different calcium and phosphorus levels. The size of the dot for each genus shows the average relative abundance. Dots represent faecal microbiomes and square dots represent lipid molecules. Transparency of the line indicates the negative logarithm of the correlation *P*-value (Spearman’s) (bottomed out at 10), the green line indicates a negative correlation, the orange line indicates a positive correlation, and the width of the line indicates a large correlation (Spearman’s)

The PCoA analysis further confirmed the significant isolation of the flora composition among the three groups (Fig. 6B). Venn diagram revealed an increase in the total number of OTUs due to changes in calcium and phosphorus levels (Fig. 6C). Phyla-level microbial relative abundance analysis showed that although the relative abundance of Firmicutes did not change and the ratio of Firmicutes/Bacteroidetes did not differ significantly, the relative abundance of Bacteroidota decreased with increasing levels of calcium and phosphorus (Fig. 6D). Family-level relative abundance (Fig. 6E) showed that Prevotellaceae, Streptococcaceae, and Veillonellaceae were negatively correlated with calcium and phosphorus concentrations. Compositional analyses at the genus level revealed significantly lower relative abundance of *Prevotella_9* in the HCAP group compared to the CON group, and significantly lower relative abundance of *Alloprevotella* compared to the LCAP group (*P* < 0.05) (Fig. 6G). LEfSe analyses identified microbial communities that significantly differed across dietary calcium and phosphorus levels. *Micrococcales* and *g_Lactococcus* were dominant in the CON group, whereas *g_Alloprevotella*, *g_Streptococcus*, and f_Streptococcaceae were dominant in the LCAP group. Tissierellales, Peptostreptococcaceae, *Terrisporobacter*, and *Prevotellaceae_NK3B31_group*, on the other hand, were the characteristic flora of the HCAP group (Fig. 6H). KEGG pathway analyses showed that, compared with the CON and LCAP groups, the HCAP group had a higher predominance in amino acid metabolism, carbohydrate metabolism, lipid metabolism, membrane transport and metabolism pathways were up-regulated in abundance, suggesting that increased calcium and phosphorus levels in the feed contributed to improved lipid metabolism (Fig. 7A). The results of short-chain fatty acid (SCFA) content showed that low calcium-phosphorus levels significantly reduced the concentrations of isobutyric acid and isovaleric acids, while high calcium-phosphorus levels reduced the concentration of acetic acid (Fig. 7B), further confirming that calcium-phosphorus levels in feeds have a significant regulatory effect on intestinal metabolites.

### Linkage between the microbiome and lipidomics contributes to the understanding of calcium-phosphorus-regulated lipid metabolism mechanisms

By Spearman’s correlation coefficient analysis, we explored the relationship between gut microbial communities and serum glycolipid metabolic indices, which were presented as a heat map (Fig. 7C). At the genus level, HDL-C showed significant negative correlations with *Lachnospiraceae_NK4A136_group*, *Selenomonas*, and *Subdoligranulum* (*P* < 0.0001 to *P* < 0.001), whereas it showed a positive correlation with *Streptococcus* (*P* < 0.05). In addition, serum TG showed negative correlation with *Alloprevotella*, *Anaerovibrio*, *Megasphaera* (*P* < 0.05) and positive correlation with *Lachnospiraceae_AC2044_group*, *Terrisporobacter* (*P* < 0.05). Through secreting metabolites into the blood, the gut microbiota is involved in the occurrence and progression of diseases [16]. Spearman correlation analysis revealed a correlation between 21 faecal OTUs and 19 significantly different lipid molecules (Fig. 7D). Significantly different lipid molecules TG (18:2_13:0_18:2), Cer (d36:0), and PE (18:1_22:1) correlated with a wide range of differentiated bacterial genera. Among them *Lachnospiraceae_NK4A136_group*, which was previously found to be negatively correlated with serum HDL-C concentration, was significantly positively correlated with the lipid molecule Cer (d46:7). *Alloprevotella*, which correlated with serum total cholesterol and HDL-C/LDL-C ratio, correlated with TG (18:1_18:1_18:2).

## Discussion

In this study, we systematically investigated for the first time the effects of different calcium and phosphorus levels in the same proportion of feed on lipid metabolism in weaned piglets. It reveals the disorder of lipid metabolism that may result from low calcium-phosphorus levels and elucidates the mechanism of how high-calcium-phosphorus feeds improve lipid metabolism by activating specific signaling pathways. Our results showed that low calcium and phosphorus diets caused significant lipid accumulation in the body and liver of piglets, which was mainly attributed to increased intestinal lipid absorption and abnormal liver lipid accumulation. This finding is consistent with the view of the intestine and liver as the main sites of lipid metabolism and emphasis the importance of regulating the lipid processing capacity of these two organs to maintain lipid homeostasis. Furthermore, our study also showed that by providing a high-calcium phosphate diet, the CAMKK2-AMPKα pathway can be activated, promoting intestinal lipid absorption and expression of transporters, increasing oxidative hydrolysis of renal lipids; At the same time reduce the synthesis of liver lipids, thus effectively improve the lipid metabolic disorder. Combined with the correlation analysis between gut microbial composition and serum lipid metabolism indexes, this study further reveals the important role of gut microbes in the regulation of lipid metabolism. The association of specific microbiota with lipid metabolism-related indicators reinforces the idea that the gut microbiota may be involved in the regulation of lipid metabolism by influencing the secretion patterns of host metabolites. In conclusion, the present study not only highlights the effects of calcium and phosphorus intake on lipid metabolism, but also reveals the potential molecular mechanisms of lipid metabolism regulation, which provides a scientific basis for the development of future nutritional intervention strategies for lipid metabolism disorders. With the further understanding of the mechanism of lipid metabolism, the regulation of calcium and phosphorus intake may become an effective way to improve the disorder of lipid metabolism and prevent related metabolic diseases.

Calcium and phosphorus are not only the most abundant minerals in mammals, but are also involved in a variety of key physiological roles, including lipid metabolism and the synthesis and maintenance of bone structure. As a major energy source and active endocrine organ in the body, lipids play a crucial role in the integrity of cell membranes, protection of vital organs, hormone synthesis, and absorption and transport of vitamins, as well as directly affecting livestock and poultry performance and meat quality [17]. In this study, we found that low calcium and phosphorus diets increased carcass TG and fat percentage, although not significantly, compared with normal calcium and phosphorus levels. This finding echoes previous studies in which calcium supplementation was found to promote muscle fat accumulation [18], whereas phosphorus supplementation showed the opposite effect [4]. When calcium and phosphorus were supplemented simultaneously, however, a reduction in muscle lipid accumulation was observed [19], which is consistent with our experimental results. Notably, adjustment of calcium and phosphorus levels in the feed significantly affected blood glucose concentrations, where high calcium and phosphorus levels significantly reduced blood glucose levels, suggesting that calcium and phosphorus supplementation may play a role in regulating glucose metabolism. Furthermore, we also observed an effect of calcium and phosphorus supplementation on the HDL-C to LDL-C ratio, consistent with Zhang et al. [20]. Although no significant changes were found in other serum lipid metabolic indexes, the decrease of HDL-C/LDL-C ratio may reflect the changes of lipid metabolism. LDL-C plays a key role in cholesterol transport and the synthesis of cell membranes and certain hormones, while HDL-C is responsible for transporting cholesterol in tissues and maintaining the stability of the cardiovascular system. Therefore, changes in the HDL-C/LDL-C ratio may affect cardiovascular health, suggesting a possible role for calcium and phosphorus in maintaining cardiovascular stability [21].

As the main site of dietary fat absorption, the intestine plays a central role in the overall lipid metabolism. Recent studies have revealed an association between dysregulation of intestinal lipid metabolism and systemic lipid metabolic diseases [22]. Nevertheless, the specific effects of dietary calcium-phosphorus ratios on intestinal lipid metabolism and their underlying mechanisms are not fully understood. The intestinal lipid accumulation, lipid absorption (CD36, FABP2, FABP3, and FABP4), lipid synthesis (DGAT1, DGAT2, ACC, and PPAR γ) gene and protein expression were detected. Here we reveal a correlation between changes in feed calcium and phosphorus levels and intestinal lipid absorption, pointing to a link between enhanced fatty acid uptake capacity and elevated calcium and phosphorus levels. Furthermore, we observed that low calcium and phosphorus levels promote abnormal accumulation of liver lipids, which results from increased lipid synthesis and inhibition of lipid transport and oxidative hydrolysis of key enzyme activities. In contrast, increased calcium and phosphorus levels mitigated this phenomenon, consistent with previous findings that altering calcium or phosphorus levels alone affects lipid accumulation [23]. These findings emphasis the important influence of the dietary calcium-phosphorus ratio on the lipid metabolism capacity of the intestine-liver axis. Previous studies on rodents [24], poultry [4], fish [25], and pigs [23] have all shown that, calcium or phosphorus supplementation alone reduced fat accumulation in the liver and intestines, and we also altered calcium and phosphorus levels in the liver to produce changes consistent with them. The occurrence of this phenomenon may be related to the fact that calcium and phosphorus levels regulate beta oxidation of fatty acids, which is the main pathway of fatty acid degradation. However, the lipid accumulation in the intestine was contrary to previous reports, and the mechanisms need to be further explored because of the paucity of studies on the effects of dietary Ca and P levels on lipid metabolism.

AMPK plays a crucial role in the maintenance of cellular energy homeostasis, not only regulating glycolipid metabolism, but also being involved in the modulation of appetite and anorexia signaling [6]. Although the inhibitory effect of calcium on endogenous lipid production in the liver by activating the AMPK pathway has been reported, its underlying molecular mechanisms are still at the forefront of exploration [7]. CAMKK2, an upstream kinase of AMPK, plays a critical role in regulating lipid metabolism by phosphorylating AMPK at the Thr172 site. Furthermore, the importance of CAMKK2’s function in regulating lipid metabolism was demonstrated by the ability of its deletion or pharmacological inhibition to reduce ab initio lipogenesis, which shows potential therapeutic value in ameliorating high-fat diet-induced fatty liver, insulin sensitivity problems [11, 12]. As previously reported, elevated levels of dietary calcium and phosphorus increase intracytoplasmic calcium ion concentrations [25, 26] and serum lipocalin concentrations [27, 28], and lipocalin induces activation of the lipocalin receptor 1 (AdipoR1), which drives CAMKK2 to increase AMPK activity by activating extracellular Ca^2+^ efflux [29]. We further hypothesized that dietary calcium and phosphorus levels might regulate intestinal-liver axis lipid metabolism through the CAMKK2/AMPK signaling pathway. We observed that low calcium-phosphorus levels decreased mRNA and protein levels of intestinal CAMKK2 and AMPKα, accompanied by a decrease in lipid deposition, while high calcium-phosphorus levels reversed this trend. This phenomenon may be related to the upregulation of AMPK activity, which enhances the uptake capacity of long-chain fatty acids (LCFA) in the gut by promoting the expression and membrane translocation of CD36 and FATP4 proteins; This leads to lipid accumulation [30]. Furthermore, we found that dietary calcium and phosphorus supplementation activated the liver CAMKK2/AMPK signaling pathway to mitigate abnormal accumulation of liver lipids by reducing lipid synthesis and enhancing lipid hydrolytic oxidation processes; This process involves the upregulation of SIRT1, a signaling molecule downstream of AMPK. Our results suggest that dietary calcium and phosphorus levels regulate intestinal-liver axis lipid metabolism through the CAMKK2/AMPK signaling pathway.

Given that Ca and P supplementation is known to influence lipid metabolism in skeletal muscle and adipose tissue [1, 18], the present study examined the effects of changes in calcium and phosphorus dietary levels on liver lipid composition, which is important for understanding the role of calcium and phosphorus in overall metabolic regulation. Our findings reveal that low calcium phosphorus intake is associated with increased levels of triglycerides (MG and TG) and certain phospholipids (PIP_2_ and SPH) and sphingolipids (GM3 and SM) in the liver; The concentrations of LPG and Ceramide (Cer) were also reduced. This finding implies that dietary calcium and phosphorus levels are negatively correlated with specific lipid classes in the liver. Through the KEGG pathway analysis of differential lipids, we further verified that these lipids are mainly involved in glyceride and glycerophospholipid metabolic pathways. The increase of glycerides, especially MG and TG, may reflect the accumulation of intermediate products during lipolysis and the enhancement of activity of TG biosynthesis pathway, this is consistent with existing studies of the effects of calcium and phosphorus supplementation alone on lipid metabolism. In addition, the up-regulation of PIP_2_ and SPH, as well as the increase in GM3 and SM, may involve changes in cell signaling, cell proliferation and apoptosis, as well as cell membrane composition and function. In particular, changes in SPH and SM implicate a potential role for sphingolipid metabolism in regulating energy homeostasis and AMPK expression. The changes of SPH and SM may affect the energy metabolism of liver by affecting the physical properties of cell membrane and cell signaling pathway [31]. In particular, SM (d18:1/16:0) was found to enhance ATP production and reduce AMPK expression by activating glycolytic pathways [32]. This provides new insights into the interaction between lipid metabolism and energy sensing.

To establish a potential link between dietary Ca, P, and the microbiome and specific lipid species, we performed a microbiome analysis of colonic contents in the LCAP, HCAP, and CON groups. We found that both the LCAP and HCAP groups increased the alpha diversity of the colonic microbiota compared to the CON group. This may reflect changes in microbial community structure leading to an increase in the diversity of harmful bacteria [33]. Indeed, a closer examination of the individual bacterial composition of LCAP and HCAP revealed an increased abundance of bacteria belonging to the Enterobacteriaceae, Streptococcaceae, and Clostridiaceae, which are families of bacteria that contain pathogenic bacteria [34]. The β-diversity analysis further revealed significant differences in microbiome structure among the three groups, consistent with previous findings [35]. Our examination of diet-related phylum abundance showed that changes in dietary calcium and phosphorus did not alter the abundance of the Firmicutes, but the abundance of the Bacteroidetes declined with increasing Ca and P levels, with the Firmicutes/Bacteroidetes ratio being lowest in the LCAP group. Further analyses of specific ASVs showed that bacteria from the Prevotellaceae and Veillonellaceae families decreased with increasing calcium and phosphorus levels, consistent with their association in obesity pathology [36, 37]. Strikingly, the significant reductions in *Alloprevotella* and *Prevotella* in the HCAP group compared with the LCAP group were associated with improvements in liver steatosis and other lipid metabolic markers. *Alloprevotella* and *Prevotella_9* belong to the genus *Mycobacterium*, which is a group of two in the genus *Prevotella*. Several reports have linked *Prevotella* to a range of diseases, including advanced liver fibrosis, cirrhosis, insulin resistance, type 2 diabetes, inflammation and obesity [38, 39]. Furthermore, high abundance of *Prevotella copri* in a porcine model was associated with elevated concentrations of obesity-related serum metabolites, which were significantly correlated with fat accumulation in pigs [40]. These results support our observations in this study. Our results provide new insights into the potential role of the gut microbiota in influencing lipid metabolism through calcium and phosphorus levels.

The endocrine function of the gut plays a key role in the regulation of lipid metabolism, in particular, short-chain fatty acid produced by the fermentation of gut microbes (SCFA) has a significant effect on lipid biosynthesis through the intestine-liver axis [41, 42]. We observed a decreasing trend in total SCFA levels in colonic contents with increasing dietary calcium and phosphorus levels. This finding echoes previous studies, which have shown that the majority of liver lipogenesis is dependent on microbial SCFA from the colon, which are key components of fatty acid biosynthesis [43]. Further analyses of specific SCFA concentrations revealed significant reductions in acetic acid concentrations in the HCAP group as well as isobutyric and isovaleric acid concentrations in the LCAP group. Acetic acid as the main energy source of the liver, isobutyric acid and isovalerate play critical roles in maintaining intestinal health, providing energy, having anti-inflammatory and immunomodulatory effects, and promoting the integrity of gut barrier function [44]. Their decline in LCAP seems to be explained by an increase in pathogenic bacteria. Collectively, our results suggest that dietary calcium and phosphorus levels may provide a feasible path to improve liver steatosis and optimize lipid metabolic indices by influencing SCFA production.

## Conclusion

**Fig. 8:**
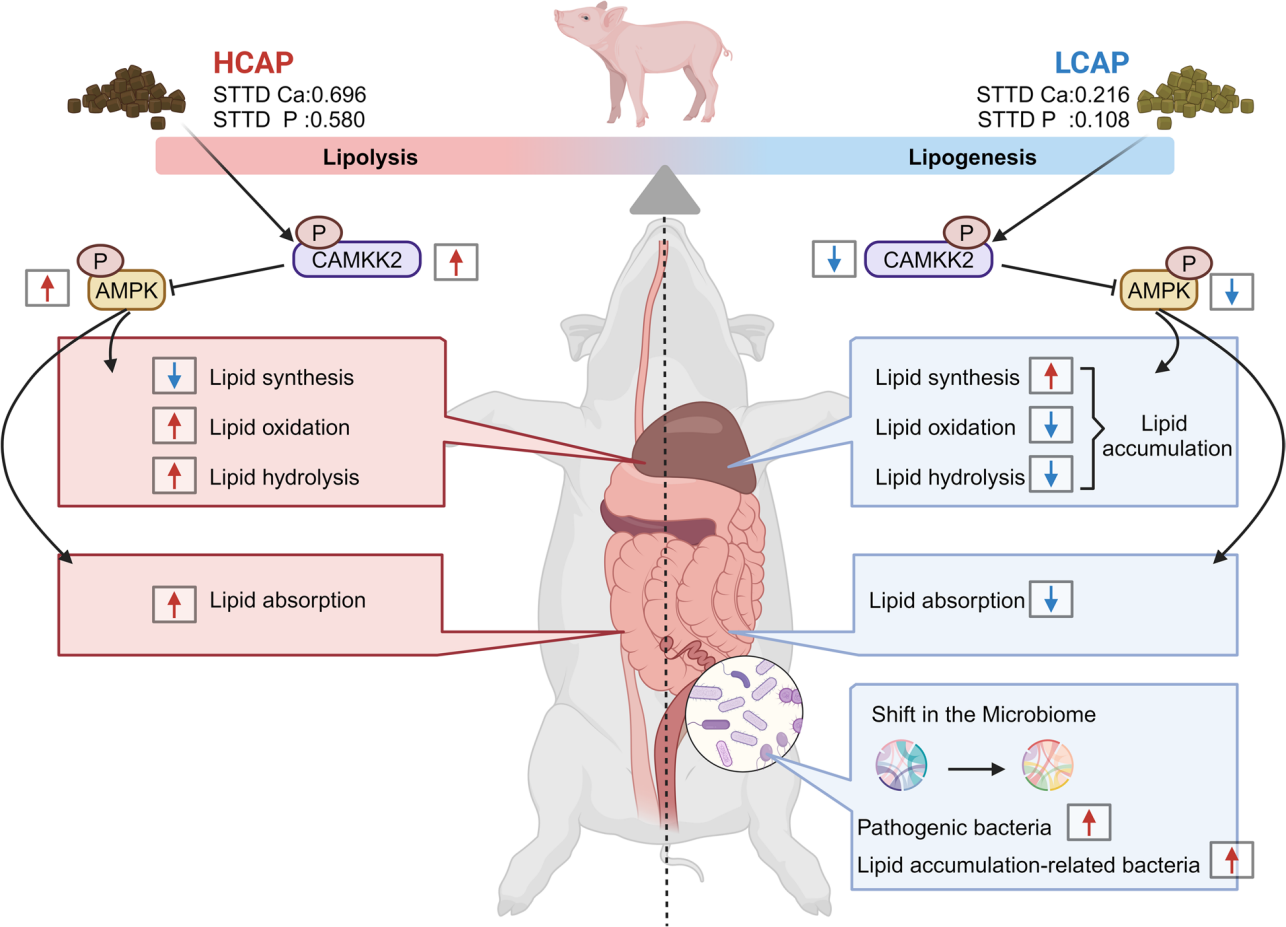
Schematic diagram of dietary calcium and phosphorus levels regulating lipid metabolism in weaned piglets. Calcium and phosphorus in the diet regulate intestinal lipid absorption and hepatic lipid metabolism through the CAMKK2/AMPK axis and alter the gut microbiota to influence lipid metabolism. Created with BioRender.com

In summary, our results indicate that when the STTD Ca: STTD P ratio is 0.216%:0.18% (LCAP), it reduces intestinal lipid absorption and leads to abnormal lipid accumulation in the liver and carcass. In contrast, supplementing calcium and phosphorus can improve lipid metabolism by promoting intestinal lipid transport and liver lipid hydrolysis and oxidation through the activation of the CAMKK2/AMPK pathway. Changes in dietary calcium and phosphorus levels can also regulate the gut microbiota. Overall, we report on the response of intestinal-liver axis lipid metabolism to dietary calcium and phosphorus levels and its regulatory mechanisms, and reveal how these changes affect liver lipid composition and the characteristics of the intestinal microbiome (Fig. 8). These findings underscore the importance of precisely managing dietary mineral levels to optimize lipid metabolism and enhance the overall health of animals.

### Supplementary Information

**Additional file 1: Table S1.** The primer sequence of qPCR.

**Additional file 2. **Uncropped and unprocessed images of the complete gel and blot in Fig. 3B, 3E, 4F and 4L.

## Data Availability

The data analyzed during the current study are available from the corresponding author on reasonable request.

## Abbreviations

ACC1: Acetyl-CoA carboxylase 1
AMPK: 5′-Adenosine monophosphate-activated protein kinase
AMPKα: AMP-activated protein kinase alpha
Ca: Calcium
CAMKK2: Calcium/calmodulin-dependent protein kinase kinase 2
CD36: Cluster of differentiation 36
Cer: Ceramide
CPT1A: Carnitine palmitoyltransferase 1a
DGAT1: Diacylglycerol O-acyltransferase 1
FABP2: Fatty acid binding protein 2
FABP3: Fatty acid binding protein 3
FASN: Fatty acid synthase
FATP4: Fatty acid transport protein 4
GLU: Glucose
GM3: Monosyalilated ganglioside M3
HDL-C: High-density lipoprotein cholesterol
LDL-C: Low-density lipoprotein cholesterol
LPG: Lysophosphatidylglycerol
MG: Monoradylglycerols
NAFLD: Nonalcoholic fatty liver disease
OTU: Operational taxonomic unit
P: Phosphorus
P-AMPKα: Phosphorylated AMPKα
P-CAMKK2: Phosphorylated CAMKK2
PIP_2_: Phosphatidylinositol 4,5-bisphosphate
PPARγ: Peroxisome proliferator-activated receptor γ
PRDM16: PR domain-containing protein 16
SCD: Stearoyl-CoA desaturase
SCFA: Short chain fatty acid
SIRT1: Sirtuin 1
SM: Sphingomyelin
SPH: Sphingosine
SREPB1C: Sterol regulatory element-binding protein 1c
STTD: Standardized total tract digestibility
TC: Total cholesterol
TG: Triglyceride
