# Supplementary material for: CAMKK2-AMPK axis endows dietary calcium and phosphorus levels with regulatory effects on lipid metabolism in weaned piglets
Source: J Anim Sci Biotechnol. 2024 Aug 5;15:105. doi: 10.1186/s40104-024-01061-0 (PMC11299266; doi:10.1186/s40104-024-01061-0)
Supplement: Supplementary file 1 — Additional file 1: Table S1. The primer sequence of qPCR. [file 40104_2024_1061_MOESM1_ESM.docx]

**Additional file 1**

**Table S1** The primer sequence of qPCR

| **Primer name** | **Sequence (5'→3')** |
| --- | --- |
| *β-actin-F* | CCACGAAACTACCTTCAACTC |
| *β-actin-R* | TGATCTCCTTCTGCATCC |
| *DGAT1-F* | AACCTGACCTACCGCGATCT |
| *DGAT1-R* | GGAAGCGGGAAAAGTTGAGC |
| *FASN-F* | TGGGCATGGTGAACTGTCTC |
| *FASN-R* | TGGGCATGGTGAACTGTCTC |
| *CD36-F* | GCATCACAGCCTACACCACAGC |
| *CD36-R* | CGAGCCAGAGATTGAACCCACATC |
| *FABP1-F* | ATCACTACCGGGTCCAAGGT |
| *FABP1-R* | CAACTGAACCACTGTCTTGACC |
| *FABP2-F* | CGGAACTGAACTCACTGGGAA |
| *FABP2-R* | CTGGACCATTTCATCCCCGA |
| *FABP3-F* | GATGACAGGAAGGTCAAGTCCA |
| *FABP3-R* | TAAGTGCGAGTGCAAACTGC |
| *FABP4-F* | AAGAAGTGGGAGTGGGCTTTGC |
| *FABP4-R* | ATTCTGGTAGCCGTGACACCTTTC |
| *ACC-F* | CGGAATATCCAGAAGGCCGA |
| *ACC-R* | CCAGTCCGATTCTTGCTCCA |
| *PPARα-F* | CAGCAATAACCCGCCTTTCG |
| *PPARα-R* | CTCCTTGTTCTGGATGCCGT |
| *PPARγ-F* | GCAGGAGCAGAGCAAAGAGGTG |
| *PPARγ-R* | GCCAGGTCGCTGTCATCTAATTCC |
| *SREBP1-F* | CACGGAGGCGAAGCTGAATA |
| *SREBP1-R* | CTGGTTGCTCTGCTGAAGGA |
| *CPT1A-R* | TTCAGTTCACGGTCACTCCG |
| *CPT1A-F* | TGGATCCCAGGAGAATCGGT |
| *ATGL-R* | CCAACGCCAAGCACATCTAC |
| *ATGL-F* | TTCACCAGGTTGAAGGAGGG |
| *PRDM16-R* | CCACAAGTCCTACACGCAGT |
| *PRDM16-F* | CGGGTAATGGTTCTTGCCCT |
| *AMPKα-F* | GGTGAAAATCGGCCACTACA |
| *AMPKα-R* | TTGCCAACCTTCACTTTGCC |
| *SIRT1-R* | GGTTAGGAGGTGAATATGCCAAG |
| *SIRT1-F* | TCTGACAAGTGAGCCAACTCT |
| *CAMKK2-R* | TTGTGGAATCTGGCTTGG |
| *CAMKK2-F* | TGGTCTGCTTCTCTGTCT |
