# Supplementary material for: CAMKK2-AMPK axis endows dietary calcium and phosphorus levels with regulatory effects on lipid metabolism in weaned piglets
Source: J Anim Sci Biotechnol. 2024 Aug 5;15:105. doi: 10.1186/s40104-024-01061-0 (PMC11299266; doi:10.1186/s40104-024-01061-0)

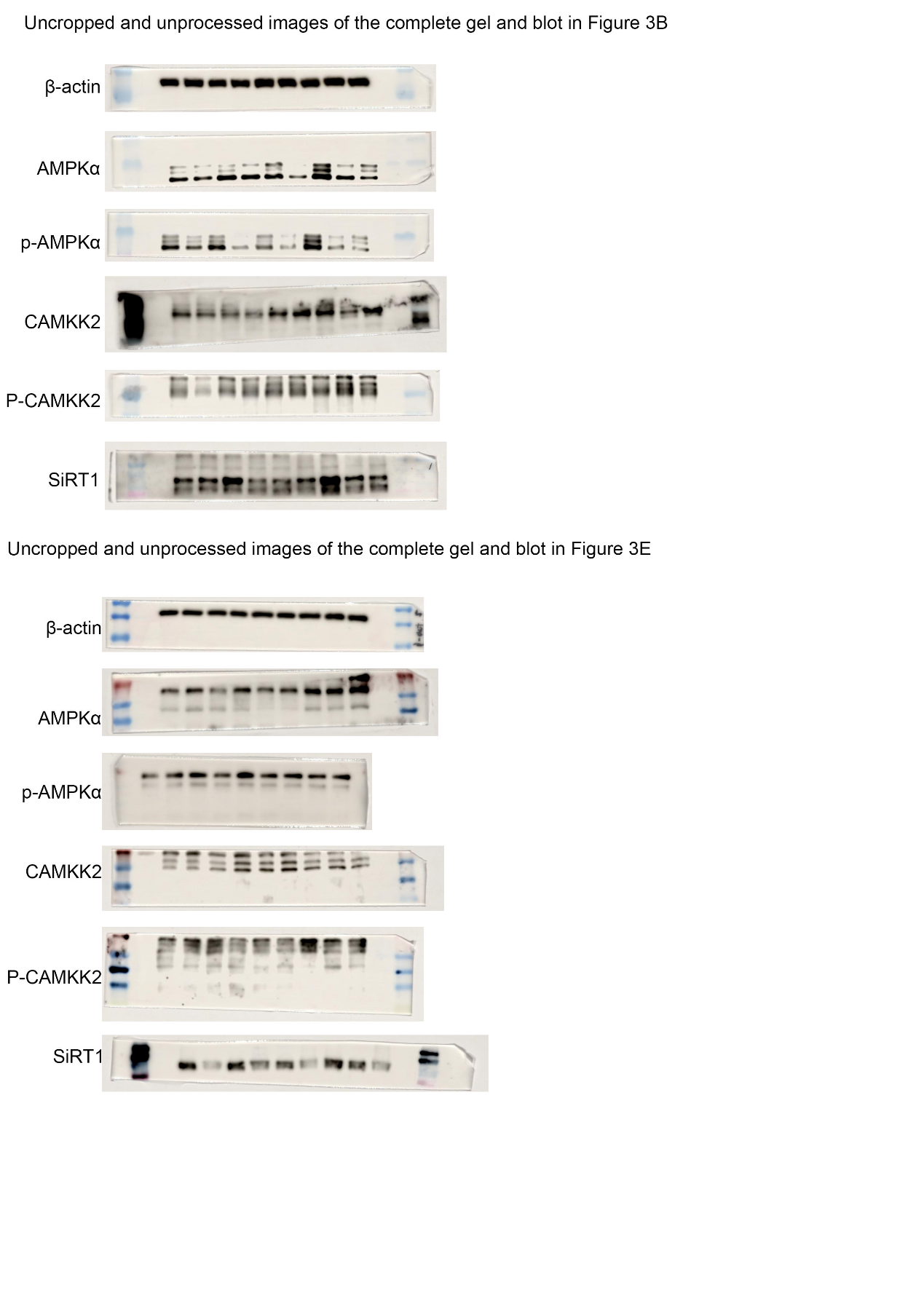
Uncropped and unprocessed images of the complete gel and blot in Figure 3B

Uncropped and unprocessed images of the complete gel and blot in Figure 3E


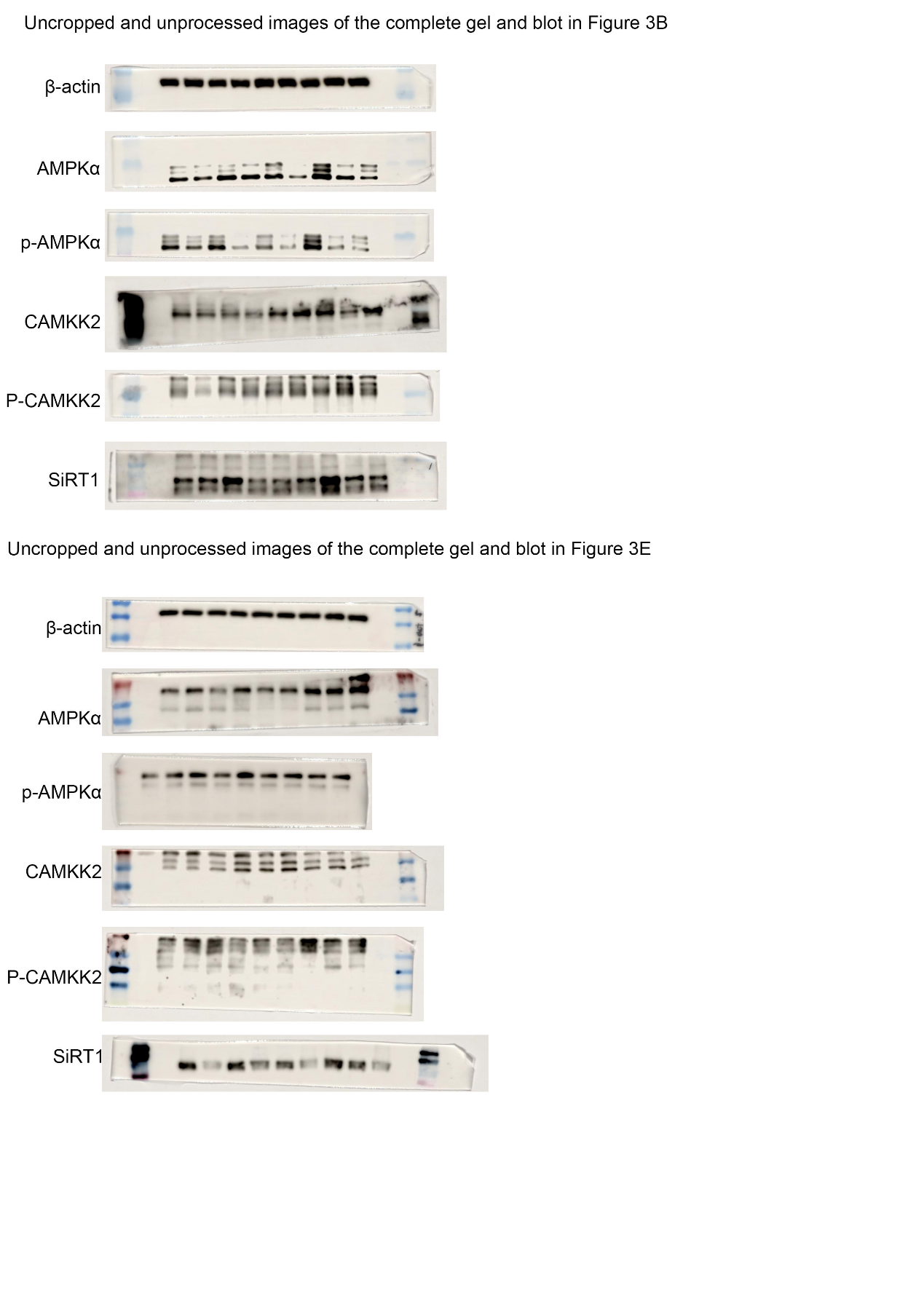


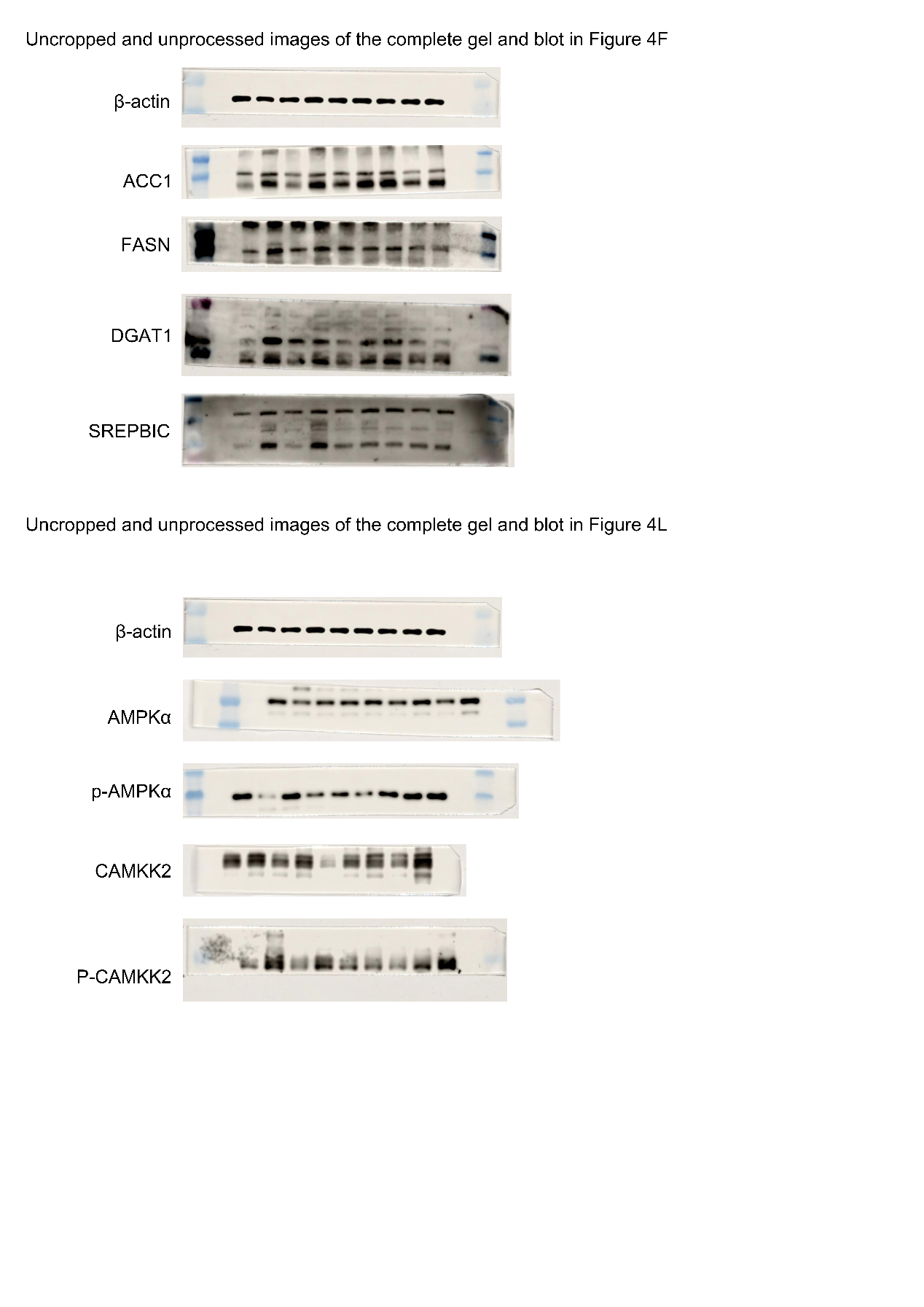
Uncropped and unprocessed images of the complete gel and blot in Figure 4F
Uncropped and unprocessed images of the complete gel and blot in Figure 4L

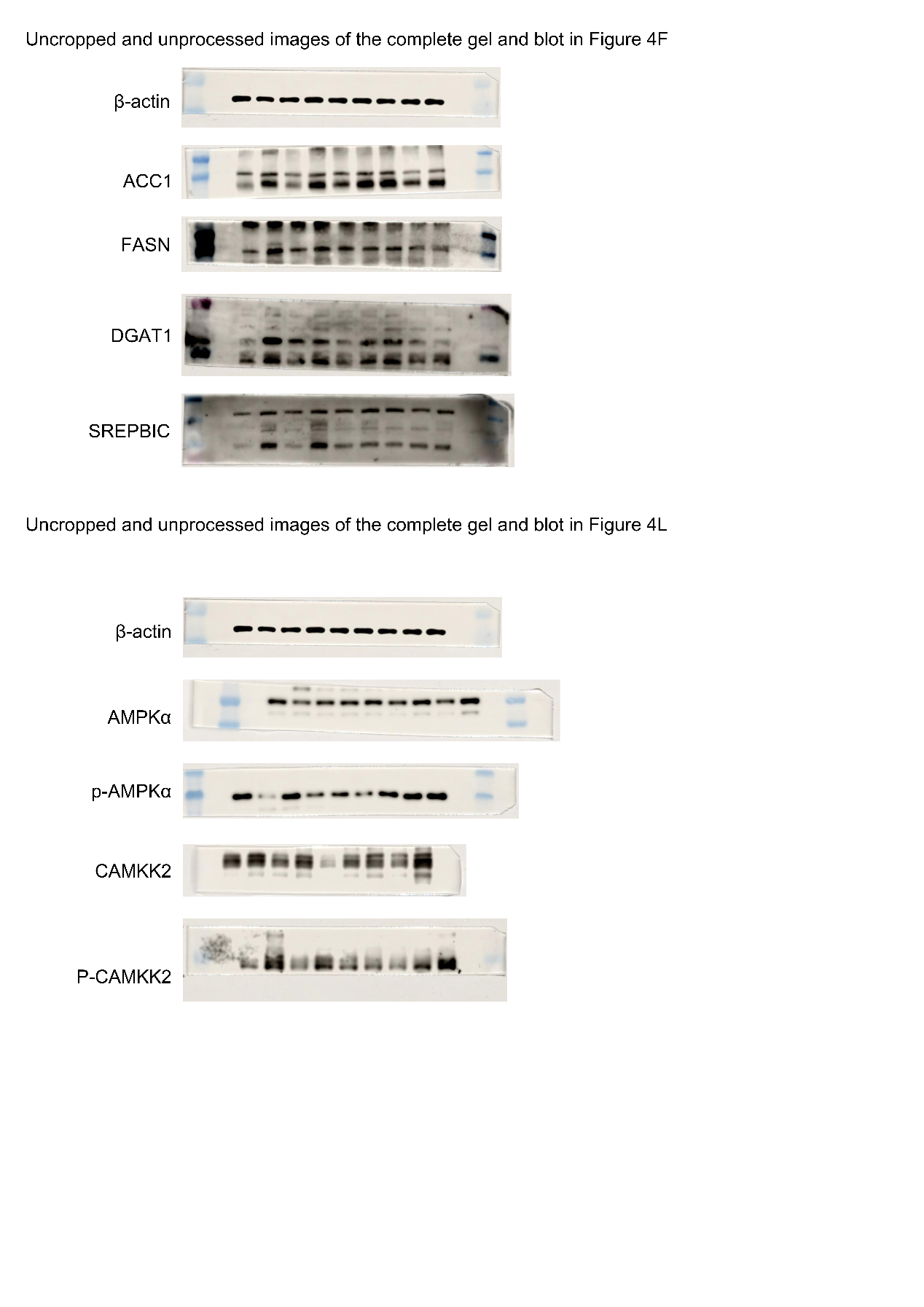

Supplement: Supplementary file 2 — Additional file 2. Uncropped and unprocessed images of the complete gel and blot in Fig. 3B, 3E, 4F and 4L. [file 40104_2024_1061_MOESM2_ESM.docx]
